# Electrophysiological Properties of Embryonic Stem Cell-Derived Neurons

**DOI:** 10.1371/journal.pone.0024169

**Published:** 2011-08-26

**Authors:** Jessica R. Risner-Janiczek, Mark A. Ungless, Meng Li

**Affiliations:** Medical Research Council, Clinical Sciences Centre, Faculty of Medicine, Imperial College, London, United Kingdom; Dalhousie University, Canada

## Abstract

In vitro generation of functional neurons from embryonic stem (ES) cells and induced pluripotent stem cells offers exciting opportunities for dissecting gene function, disease modelling, and therapeutic drug screening. To realize the potential of stem cells in these biomedical applications, a complete understanding of the cell models of interest is required. While rapid advances have been made in developing the technologies for directed induction of defined neuronal subtypes, most published works focus on the molecular characterization of the derived neural cultures. To characterize the functional properties of these neural cultures, we utilized an ES cell model that gave rise to neurons expressing the green fluorescent protein (GFP) and conducted targeted whole-cell electrophysiological recordings from ES cell-derived neurons. Current-clamp recordings revealed that most neurons could fire single overshooting action potentials; in some cases multiple action potentials could be evoked by depolarization, or occurred spontaneously. Voltage-clamp recordings revealed that neurons exhibited neuronal-like currents, including an outward current typical of a delayed rectifier potassium conductance and a fast-activating, fast-inactivating inward current, typical of a sodium conductance. Taken together, these results indicate that ES cell-derived GFP^+^ neurons in culture display functional neuronal properties even at early stages of differentiation.

## Introduction

Embryonic stem (ES) cells retain the ability to provide an unlimited supply of all cell types. Much research has focused on transplanting ES cell-derived neural cells in animal models of neurological diseases and examining their potential in expressing appropriate regional markers characteristic to the surrounding host tissue. These studies indicate that ES cell-derived neural progeny are able to integrate into the host brain and undergo further differentiation [1]. Some studies have also begun to characterize the functional properties of ES cell-derived neurons using electrophysiology [1]–[3]. These studies show expression and maturation of functional excitatory and inhibitory synaptic connections, action potential firing, and isolation of voltage-gated currents (e.g., K^+^ and Na^+^) in the transplanted neurons.

The unambiguous identification of ES cell-derived neurons in the above studies often employ genetic marking of ES cell lines with a reporter, such as beta-gal or green fluorescent protein (GFP) expressed constitutively and ubiquitously [4]–[6], or restrictively in ES cell-derived neurons [7]–[9]. The latter was achieved by knock-in of the GFP gene into the tau (*mapt*) locus (tau-GFP) by homologous recombination. As tau is a microtubule-binding protein, GFP is expressed in the soma, dendrites, and axons of all differentiated, phenotypically normal neurons [7], allowing for the selective visualization of ES cell-derived neurons in culture and/or within host tissue following transplantation. Indeed, various studies have utilized the targeted tau-GFP ES cell lines in order to gain further understanding the fundamentals of neuronal development as well as the intricacies involved in cell replacement therapies [1]–[3], [7], [8].

With the increasing interest in using ES cell- and induced pluripotent stem (iPS) cell-derived neurons as platforms for drug screening and disease modelling, it is necessary to gain a greater understanding of the electrophysiological properties of these neurons in culture. Comparisons of the electrophysiological properties of ES cell-derived neurons in culture with data acquired post-grafting would provide insight into whether ES cell-derived neurons develop their functional properties in an autonomous manner or if they require additional stimuli provided by the transplant environment. Furthermore, establishing a functional phenotypic profile of in vitro derived neurons will provide a complimentary system for conducting studies prior to transplantation.

Here we examine the electrophysiological properties of neurons derived from mouse TK23 ES cells expressing GFP under the control of the endogenous tau promoter. We found that GFP^+^ TK23 progeny displayed neuronal-like characteristics including the expression of voltage-gated currents and the ability to fire action potentials. These properties were similar to those seen in early stages of transplantation [1].

## Materials and Methods

### ES cell maintenance and neural differentiation

TK23, a mouse ES cell line carrying a GFP reporter targeted to the tau (*mapt)* locus, was generated previously [9], [10] using the targeting vector from Tucker et al. [7]. These cells were maintained on gelatine-coated plates in GMEM (Glasgow Minimum Essential Media; Invitrogen, 21710-025) supplemented with 10% FCS (Biosera, S1810-500), 2 mM L-glutamine (Invitrogen, 25030), 100 µM MEM nonessential amino acids (Invitrogen, 11140), 1 mM sodium pyruvate (Invitrogen, 11360), 50 µM 2-mercaptoethanol (Invitrogen, 31350-010), and leukemia inhibitory factor (LIF). Neuronal differentiation was carried out using a monolayer protocol as described in Ying et al. [9]. Briefly, ES cells were trypsinized and re-plated onto gelatinized tissue culture plates in retinol-free N2B27 media. After culturing overnight, fresh N2B27 media was applied to the cultures and media was refreshed every other day. After assessing cell morphology and confluency, cultures were re-plated (typically day 4) onto round coverslips coated with poly-L-lysine (Sigma, P4707) and laminin (Sigma, L2020) and placed into 6-well culture plates with N2B27 media. Electrophysiological assessment of TK23-derived GFP^+^ neurons was carried out in day 9–16 cultures.

### Electrophysiology

Coverslips of day 9–16 cultures were placed onto a recording chamber and viewed using an Olympus BX51WI microscope with a 40x water immersion lens and DIC (differential interference contrast) optics. Cells were bathed in a solution containing (in mM): 140 NaCl, 3.5 KCl, 1.25 NaH_2_PO_4_, 2 CaCl_2_, 1 MgCl_2_, 10 Glucose, and 10 HEPES. For whole-cell electrophysiological recordings, low resistance recording pipettes (7–12 MΩ) were pulled from capillary glass (Harvard Apparatus Ltd.) and coated with ski wax to reduce pipette capacitance. Recording pipettes were filled with a solution containing (in mM): 140 K-gluconate, 5 NaCl, 2 Mg-ATP, 0.5 LiGTP, 0.1 CaCl_2_, 1 MgCl_2_, 1 ethylene glycol-bis (b-aminoethyl ether) -*N,N,N*',*N*'-tetraacetic acid (EGTA), and 10 HEPES. Osmolarity and pH of both solutions were adjusted before experiments. Prior to recording from differentiated cells, GFP positive neurons were identified and targeted for recording using fluorescence via a GFP selective filter (X-Cite series 120, EXFO). Data were acquired at room temperature (20–22°C) using an Axon Multiclamp 700B amplifier and a Digidata 1440a acquisition system, with pClamp 10 software (Molecular Devices). Data analysis was carried out using Clampfit 10.2 software (Axon Instruments), OriginPro 8.1 (OriginLab Corporation), and Spike2v5 software (Cambridge Electronic Design). Data are presented as the mean ± standard error of the mean. In order to determine the activation and range for the K^+^ and Na^+^ conductances, we used a Boltzmann equation of the type
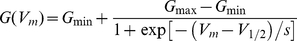
(1)where *Gmax* and *Gmin* represent the maximum and minimum conductances respectively; *V_1/2_* is the half-maximal voltage, and *s* represents the slope factor. Statistical analysis was performed using either Chi-Square tests or Student t-tests (GraphPad Prism, GraphPad Software, San Diego, CA) and significance was noted when p<0.05.

## Results

### Generation of TK23 ES cell-derived neurons

**Figure 1:**
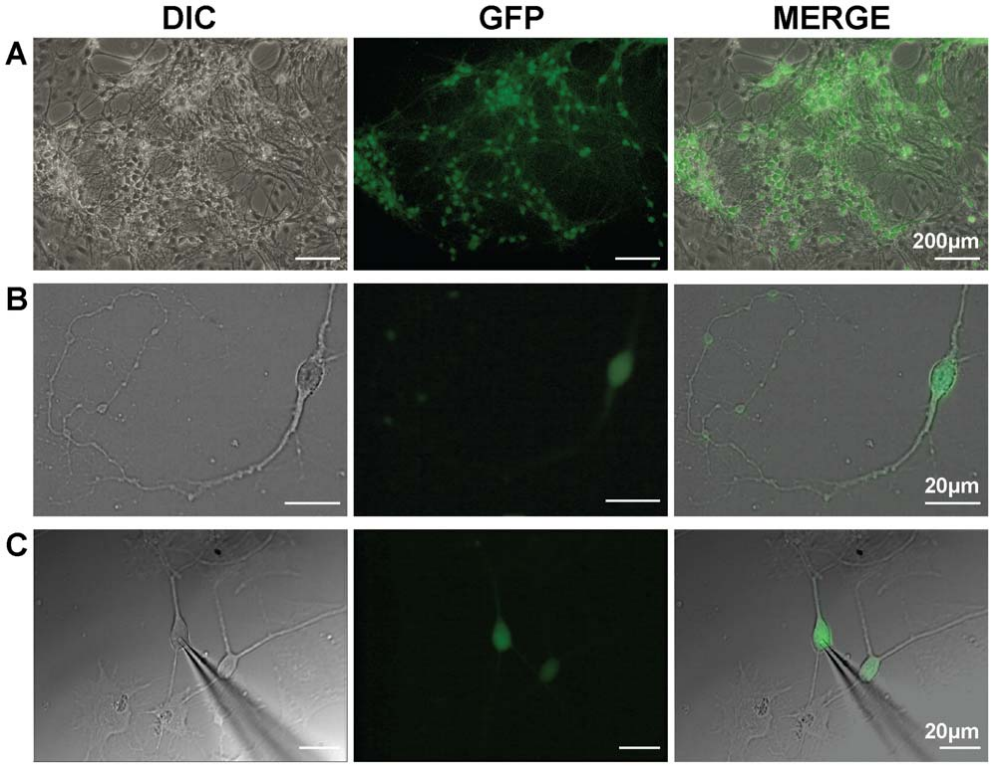
TK23 embryonic stem cells differentiate into neurons expressing GFP. **A**, TK23 cell cultures containing GFP^+^ neurons. **B**, Higher magnification image of a single GFP positive neuron, showing extensive processes. **C**, Glass microelectrode recording from a single neuron in the whole-cell configuration. DIC  =  differential image contrast; GFP  =  green fluorescent protein.

In this study, we demonstrate the efficient generation of neurons from ES cells via the monolayer differentiation protocol [9]. Neural induction and neuronal maturation of ES cells were carried out for up to 16 days. We used the TK23 ES cells, which exhibit neuronal expression of GFP under the control of the endogenous tau promoter (Fig. 1 A–C). GFP^+^ neurons were abundant within the cultures and most had small cell bodies. These cells were also multipolar, often exhibiting lengthy processes (Fig. 1 B, C).

### Passive membrane properties of TK23 ES cell-derived neurons

After reliable monolayer differentiation of TK23 cells was established, we next wanted to examine the electrophysiological properties of these neurons. In these cells, GFP fluorescence was strong and stable, allowing for easy identification of neurons in culture and targeting with a recording electrode (Fig. 1C).

We conducted whole-cell recordings from day 9 – 16 post differentiation. The neurons examined had negative resting membrane potentials that ranged from −10 to −45 mV (mean ± sem; −26±2.6 mV; n = 18). We calculated the input resistance of these cells using a 5 mV voltage step applied as a prepulse step during the voltage protocol. These calculations revealed relatively large input resistance values in these cells (1340±155 MΩ; n = 36) when compared to values reported for other neuronal groups [11], [12]. Our input resistance values were similar, however, to those reported for transplanted ES cell derivatives and directly reprogrammed neurons from fibroblasts [3], [13]. Whole-cell capacitance values were determined from the compensation values obtained during whole-cell recording using the Axon Multiclamp 700B amplifier (Molecular Devices). The observed whole-cell capacitance values were small (12±5.6 pF; n = 36), consistent with small cell size. Although these capacitance values were smaller than those reported in *ex vivo* neurons [14], [15], they were consistent with capacitance values observed in other stem cell-derived neurons during the first week of culture/transplantation [1], [13], [16].

### Action potential firing characteristics of TK23 ES cell-derived neurons

**Figure 2:**
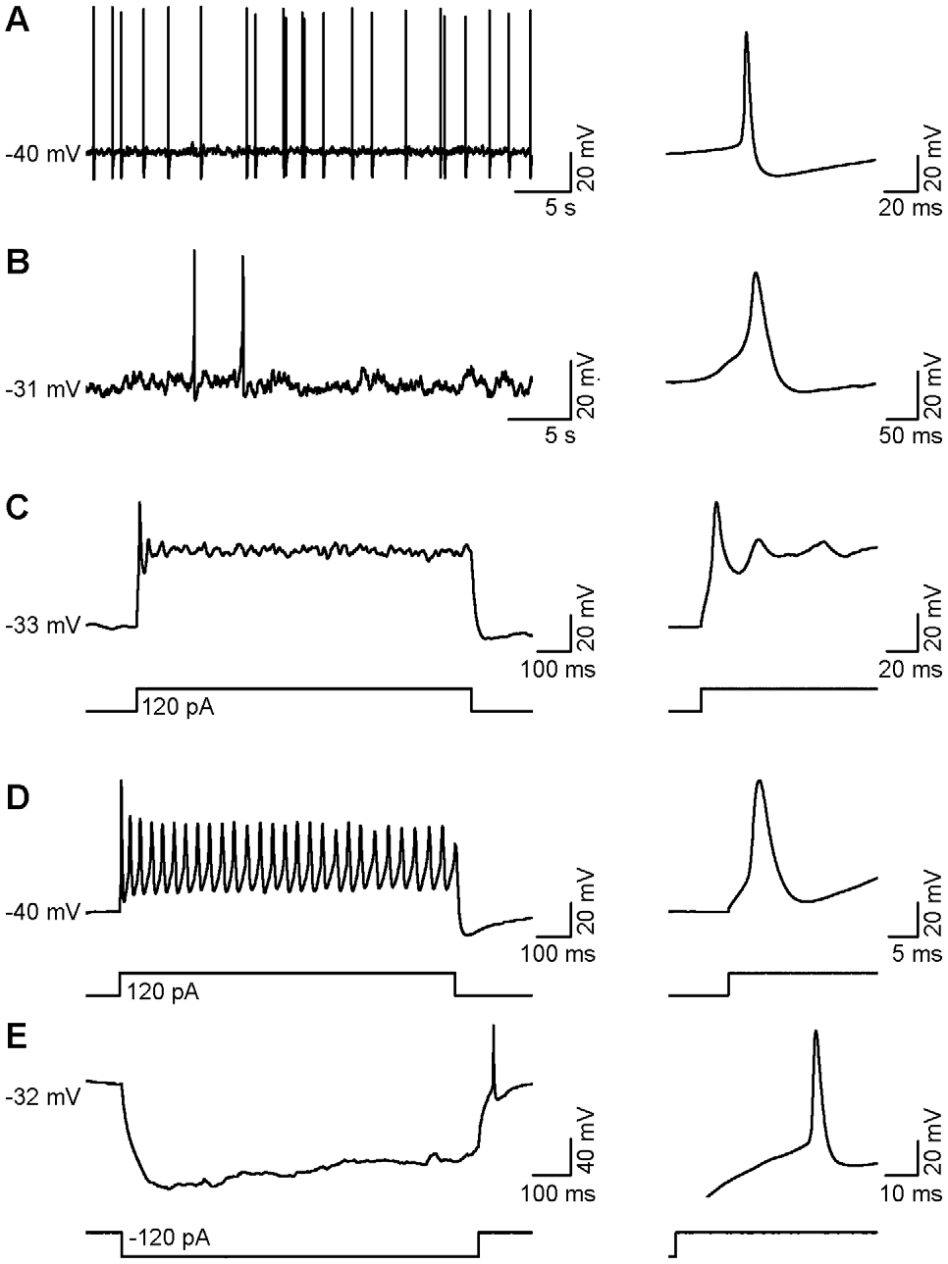
Whole-cell recordings from individual GFP^+^ TK23 cells in current-clamp mode. **A, B**, Examples of spontaneous action potential activity and averaged waveforms from two different neurons. **C**, Example of an evoked single action potential and the same spike at higher temporal resolution. **D**, Example of multiple evoked action potentials and higher temporal resolution of the first action potential. **E**, A hyperpolarizing pulse showing a depolarizing sag followed by a single rebound action potential and the same action potential at higher temporal resolution.

33% of neurons that were examined in current-clamp mode (n = 6/18) exhibited spontaneous action potentials (Fig. 2 A, B). In all cases, single overshooting action potentials were generated in response to a depolarizing current pulse (Fig. 2C), while in two neurons (2/18), multiple action potentials were generated in response to the same stimulus (Fig. 2D). Hyperpolarizing current pulses were also applied to these cells and 44% (n = 8/18) fired rebound action potentials at the end of each pulse (Fig. 2E).

As all neurons examined in current-clamp mode fired action potentials in response to a stimulus (n = 18), we decided to further analyze these individual action potential characteristics: amplitude (threshold to peak), afterhyperpolarization amplitude, and action potential width. Evoked action potentials displayed amplitudes that ranged from 15 to 93 mV (42±5.2 mV; n = 18) with afterhyperpolarization amplitudes ranging from 1.7 to 15 mV (5.4±0.9 mV; n = 18). We measured the action potential width at the half-maximal amplitude (threshold to peak), which ranged from 3.9 to 22 ms (11±1.3 ms; n = 18).

### Voltage-clamp analysis of TK23 ES cell-derived neurons

**Figure 3:**
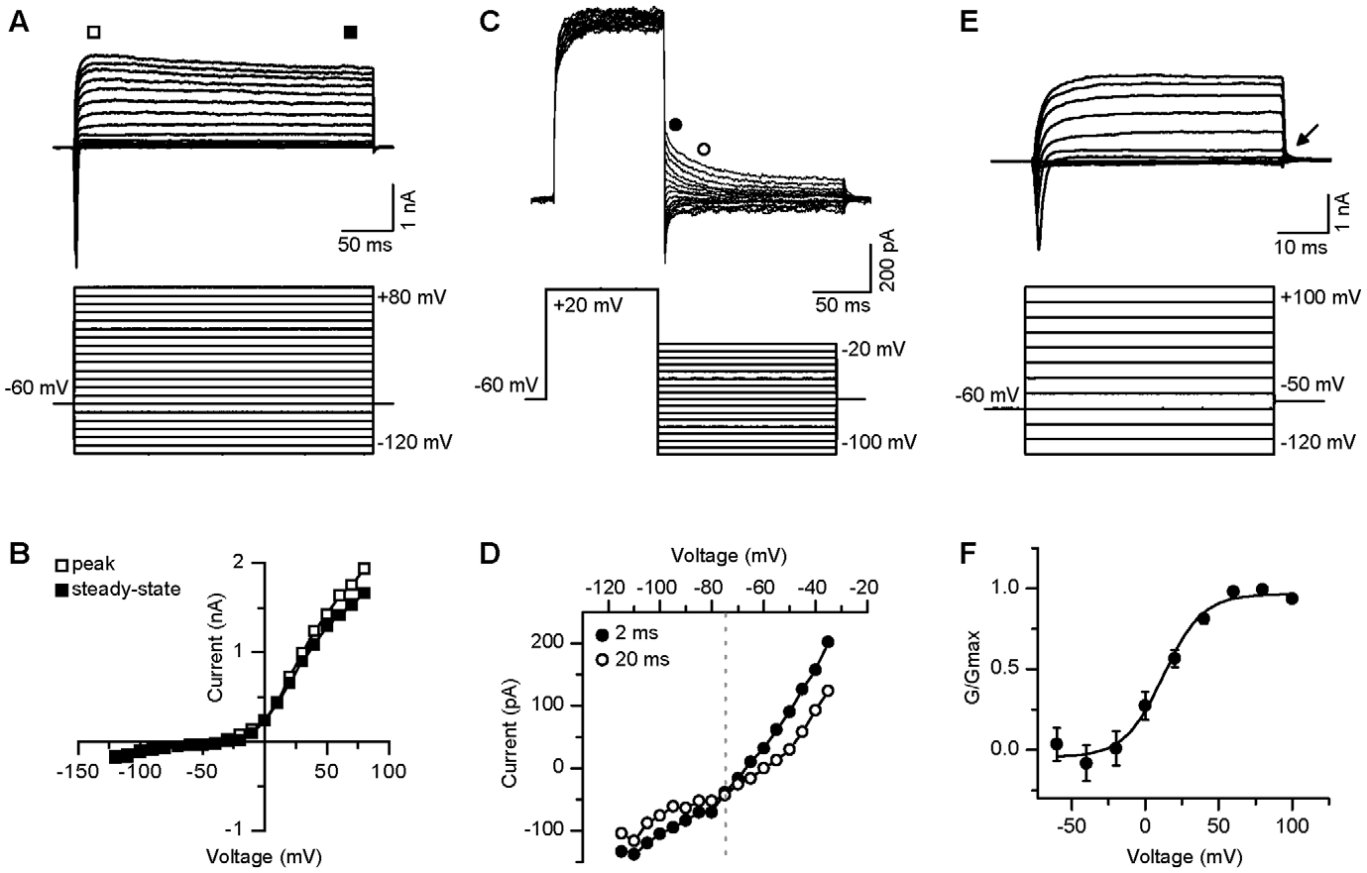
Voltage-gated potassium currents from individual GFP^+^ TK23 cells. **A,** Representative example of delayed rectifier current (upper panel) evoked by depolarizing current steps (lower panel; steps range from −120 mV to +80 mV; Vm = −60 mV). **B**, Peak (open square) and steady-state (filled square) current-voltage plot for the cell shown in **A**. **C,** Representative example of the protocol used to determine K^+^ reversal potential. K^+^ current in upper panel, protocol in lower panel (steps range from −100 mV to −20 mV; pre-pulse to +20 mV; Vm = −60 mV). **D**, Current-voltage relationship from data in **C**, illustrating values taken at 2 ms (filled circle) and 20 ms (open circle) after the end of the conditioning pulse to +20 mV. Reversal potential is noted as the point at which the two lines intersect (dashed gray line). **E,** Another example of delayed rectifier current (upper panel) evoked by short depolarizing current steps followed by a step to −50 mV for use in calculating K^+^ activation (lower panel; steps range from −120 mV to +100 mV; tail current step to −50 mV (arrow); Vm = −60 mV). **F**, Average (±sem) activation curve for all neurons (n = 35). Data obtained from the tail current (arrow in **E**), were used for the calculation of K^+^ channel activation.

We next assessed the current properties in TK23 ES cell-derived neurons. The cells were held at −60 mV and voltage-gated currents were recorded in voltage-clamp mode. All cells examined displayed neuronal-like currents. In response to incremental voltage steps from −120 mV to +80 mV (10 mV increments), all neurons displayed a large outward current with little or no inactivation, typical of a delayed rectifier K^+^ current (Fig. 3A, B). This protocol allowed us to determine the peak amplitude of these large outward K^+^ currents. The current had a mean peak amplitude at +80 mV of 2.0±0.18 nA (n = 36).

We next determined the K^+^ reversal potential by using a protocol designed to account for potential leak currents [17], [18]. Using this protocol, we first applied a depolarizing step to +20 mV to activate the K^+^ current. Following this conditioning step, we applied a family of hyperpolarizing steps from −100 to −20 mV in 5 mV increments. We plotted the data obtained at both 2 and 20 ms after the end of the conditioning step against the hyperpolarizing step potentials. The values at the point of intersection of these two lines were used to determine a mean reversal potential of −78±1.5 mV (n = 26; Fig. 3C, D). The mean reversal potential observed here was similar to values reported in other neuronal populations when a similar protocol was used [17], [18].

In addition, we examined the activation range for the observed K^+^ currents by measuring the tail currents at −50 mV following 20 mV steps ranging from −120 mV to +100 mV (Fig. 3E). This method for measuring the K+ current activation helped to reduce contamination from open channel rectification. The recorded tail current values were divided by the driving force to calculate conductance. Average K^+^ conductances were plotted as a function of prepulse potential (Fig. 3F) and fit with a first order Boltzmann equation (see methods). The V_1/2_ of K^+^ activation was 16±2.4 mV with a slope of 14±45.0 mV (n = 35) and the mean maximal conductance was 1.1±0.6 nS (n = 35).

**Figure 4:**
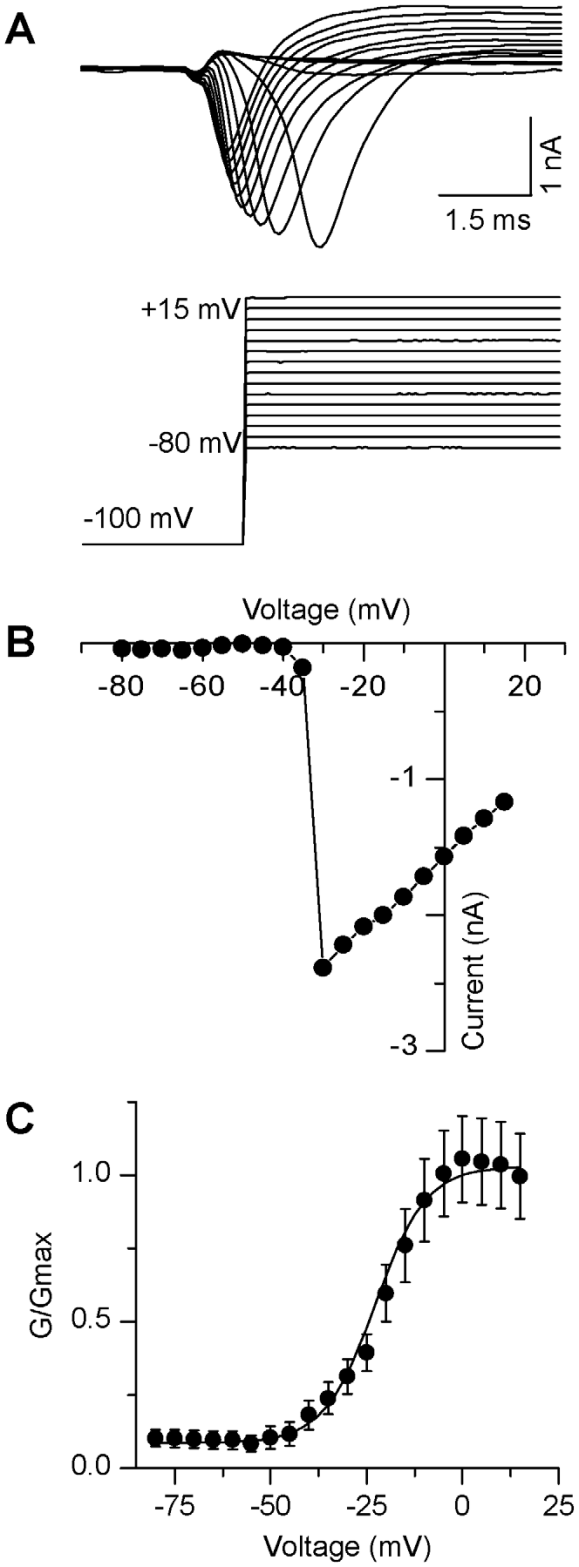
Voltage-gated sodium currents from individual GFP^+^ TK23 cells. **A,** Representative example of fast-activating, fast-inactivating inward current (upper panel) evoked by depolarizing current steps (lower panel; steps range from −80 mV to +15 mV; pre-pulse to −120 mV; Vm = −60 mV). **B**, Peak current-voltage plot for the cell shown in **A**. **C**, Average (±sem) activation curve for all neurons (n = 36).

We also observed fast-activating, fast-inactivating inward currents, which were typical of neuronal Na^+^ currents (Fig. 4A). Following a 100 ms hyperpolarizing prepulse to relieve Na^+^ channel inactivation, voltage steps ranging from −80 to +15 mV at 5 mV intervals evoked inward currents with a mean amplitude of −850±120 pA (n = 36; Fig. 4A). Na^+^ currents typically activated at −33 mV, as indicated by the Na^+^ current-voltage relationship (Fig. 4B). The V_1/2_ of activation of Na^+^ currents was also calculated and fit with a first order Boltzmann equation (see methods): −20±1.7 mV with a slope of 2.8±0.3 mV (n = 36). These cells exhibited Na^+^ conductance values of 8.9±6.9 nS (n = 36; Fig. 4C), which were obtained using a Na^+^ reversal potential value of 85 mV (derived from the Nernst equation).

Because we recorded from cells over a 7-day period, we next examined whether there were significant changes in our data over time in culture. Due to our relatively small sample size of neurons that exhibited spontaneous activity, we pooled our data into two groups: early (d9-13; n = 10) vs. late (d14–16; n = 8) in culture. Firstly, we examined the proportion of neurons firing either single, multiple, or spontaneous action potentials, and found that there was no significant difference in the action potential firing pattern over time (p>0.05). Upon examination of the current- and voltage-clamp data, we found that most properties did not change over the course of differentiation, with a few exceptions. Action potential width was found to decrease with time in culture (early: 13±1.7 ms, n = 10; late: 7.6±1.3 ms, n = 8, p<0.05). Likewise, both the Na^+^ current amplitude and Na^+^ conductance, decreased over time in culture. Na^+^ current amplitude decreased from 1.1±0.2 nA (n = 19) to 0.568±0.1 nA (n = 17; p<0.05), while the Na^+^ channel conductance decreased from 9.7±1.7 nS (n = 19) to 5.5±0.7 nS (n = 17, p<0.05). In addition, the V_1/2_ values of the K^+^ current became more positive over time (early: 9.0±2.7 mV, n = 7; late: 24±3.0 mV, n = 14). It is possible that the observed changes in voltage-gated characteristics may contribute to the decrease in action potential width, which occurred later in differentiation.

## Discussion

In this study we have shown that TK23 ES cells expressing tau-GFP can differentiate into cells that exhibit functional neuronal properties. All GFP^+^ neurons had a negative resting membrane potential and fired an action potential in response to a depolarizing stimulus. Some could fire multiple action potentials, either spontaneously or in response to a depolarizing stimulus. Recordings in voltage-clamp mode showed that all GFP^+^ neurons express currents that resemble voltage-gated K^+^ and Na^+^ currents. Though we did not examine these currents using pharmacology, we find the outward currents resemble TEA-sensitive delayed-rectifier K^+^ currents and TTX-sensitive Na^+^ currents reported in other neuronal populations [19]–[21]. These findings indicate that neurons derived from TK23 ES cells exhibit functional neuron-like characteristics, even when they remain in cell culture.

It should be noted that these neuronal properties were generally quite immature. Most neurons typically only fired a single action potential in response to a prolonged depolarization, and the voltage-gated currents seen under voltage-clamp mode were relatively small compared to mature neuronal currents [17], [22], [23]. It may be that the necessary factors for full functional maturation are limited in the mono-culture system or that the cells simply require more time to mature. Consistent with this later possibility, tau-GFP neurons used in transplant models exhibit similar single-action potential firing when examined at roughly equivalent time points [1]. Interestingly, when these cells were examined at later time points they were more likely to exhibit multiple action potentials in response to depolarization and they often fired spontaneously [1], [2].

To date, most studies have concentrated on discovering the various molecular markers that are expressed in derived neurons, and have not focused on the functional aspect of these neurons throughout differentiation. The neuronal-specific expression of GFP allows for targeted recordings of ES cell-derived neurons in cultures and/or transplant models to assess their electrophysiological properties, including potential synaptic integration [2]. The finding that ES cell-derived GFP^+^ neurons exhibit functional properties in culture offers opportunities for investigating the effects of manipulation-based alterations in the electrophysiological properties of neurons as part of a functional screening of gene expression and external drugs/reagents. Although the standard monolayer differentiation protocol used in this study primarily gives rise to GABAergic neurons, this model can be extended to other neuronal subtypes, such as midbrain dopamine neurons. These fate determination changes can occur when the cultures are stimulated with the appropriate inductive molecules (e.g., Shh and FGF8) [24], [25] in combination with the use of a subtype-specific reporter model (e.g., Pitx3-GFP) [26]. The combination of neuron-specific GFP expression and the availability of a wide array of external manipulations make this preparation a powerful model for the assessment of the functional characteristics of derived neurons in culture before, or alongside, transplantation studies.
